# Plag1 Regulates Sensorimotor Modulation in Zebrafish

**DOI:** 10.1002/jdn.70185

**Published:** 2026-10-02

**Authors:** Jemma G. Gasperoni, Jarrad N. Fuller, April L. Lewis, Travis K. Johnson, Bert De Groef, Sebastian Dworkin

**Affiliations:** ^1^ Department of Microbiology, Anatomy, Physiology and Pharmacology, and La Trobe Institute for Molecular Science La Trobe University Bundoora Victoria Australia; ^2^ Department of Biochemistry and Chemistry, and La Trobe Institute for Molecular Science La Trobe University Bundoora Victoria Australia

**Keywords:** anxiety, behaviour, neurodevelopment, Plag1, zebrafish

## Abstract

The zinc finger transcription factor *PLAG1* has been implicated in multiple diverse cellular processes, yet its role in the onset of vertebrate neurodevelopment and behaviour remains largely unexplored. Notably, dysregulation of *PLAG1* has been associated with Silver–Russell syndrome (SRS) in humans, a growth disorder often presenting with neurodevelopmental delay and craniofacial abnormalities. Here, we investigate the function of *plag1* in zebrafish embryogenesis, focusing on behavioural phenotypes, neural development and craniofacial morphogenesis, as a potential novel model of SRS. Using antisense morpholino‐mediated knockdown, we assessed the impact of *plag1* loss on brain architecture, motor neuron development, craniofacial patterning and stimulus‐evoked behavioural responses. While *plag1*‐deficient embryos displayed pronounced locomotor defects when housed in groups, these phenotypes were not recapitulated under isolated, individual tracking conditions, suggesting a role for *plag1* in modulating specific behavioural outputs. Morphological analyses revealed no overt disruptions to gross brain structure or primary motor neuron patterning. However, quantitation of craniofacial skeleton formation identified subtle abnormalities in jaw and cranial cartilage development, consistent with features observed in SRS. Furthermore, *plag1* morphants exhibited significantly altered responses to sensory stimuli, implicating *plag1* in the regulation of sensorimotor integration during early development. Collectively, these findings uncover a novel role for *plag1* in early neurobehavioural modulation and craniofacial patterning, phenocopying multiple features of SRS, and highlight the importance of environmental and social context in the phenotypic interpretation of neurodevelopmental gene function.

## Graphical abstract

PLAG1 regulates early neurobehavioural development in zebrafish. Reducing *plag1* alters craniofacial patterning, increases thigmotaxis and changes responses to light and acoustic stimuli, while gross brain structure and motor neuron development remain largely intact. These findings identify PLAG1 as a modulator of development and behaviour relevant to human Silver–Russell syndrome.

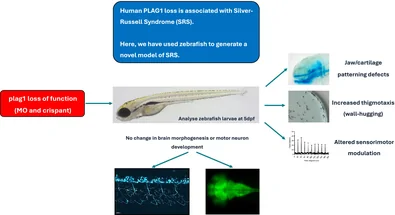

## 1 Introduction

Elucidating the genetic underpinnings of brain development and behaviour is a central challenge in developmental neuroscience and behavioural genetics. Among the candidate regulators of these complex processes is the zinc finger transcription factor *PLAG1*, which has been shown to play roles in growth and developmental regulation in murine models (Gasperoni et al. 2024; Adnani et al. 2018; Sakai et al. 2019; Juma et al. 2018; Juma et al. 2016) However, its specific functions in neural development and behavioural modulation remain largely undefined.

The zebrafish ( *Danio rerio* ) has emerged as a powerful vertebrate model for dissecting the genetic control of development, particularly within the nervous system (Dworkin et al. 2012; Dworkin et al. 2014; Dworkin et al. 2007). Its optical transparency, high fecundity and well‐characterised neuroanatomy, coupled with a high degree of evolutionary conservation with human genes, make it ideally suited to studying conserved regulators of neurodevelopment and behaviour. Additionally, the zebrafish brain is structurally comprised of discrete anatomical regions homologous to the mammalian forebrain, midbrain and hindbrain and supports a broad repertoire of quantifiable behaviours—including those relevant to human neurological conditions (Mrinalini et al. 2023). Lastly, human and zebrafish *Plag1* are well conserved, sharing approximately 56% amino acid identity across the full‐length protein. Importantly, conservation is particularly strong within the zinc‐finger DNA‐binding domain, including complete conservation of the amino acid residues predicted to mediate key DNA contacts and determine sequence specificity.

Although *plag1* expression has previously been detected in the zebrafish embryonic brain (Pendeville et al. 2006), its functional role has not been experimentally addressed. This gap is particularly noteworthy considering recent findings implicating *PLAG1* dysregulation in Silver–Russell syndrome (SRS), a congenital disorder characterised by intrauterine and postnatal growth restriction, craniofacial anomalies and variable cognitive and behavioural deficits (Burgevin et al. 2023; Dong et al. 2023; Wu et al. 2024). Given the known limitations of mammalian knockout (KO) models—where genetic compensation frequently obscures phenotypic consequences (Rossi et al. 2015; El‐Brolosy and Stainier 2017)—the zebrafish offers a tractable alternative for titrating gene function without eliciting compensatory buffering.

To date, only a single descriptive study has reported *plag1* expression in zebrafish, detailing spatiotemporal mRNA dynamics between 1 hpf and 103 dpf, with particular emphasis on the 20‐somite to 48 hpf stages and reaching a plateau at approximately 5 dpf (Pendeville et al. 2006). This work showed strong expression of *plag1* in discrete proliferative regions, particularly the ventricular zones of the brain, developing pharyngeal arches, pectoral fin buds and axial vasculature. However, no functional studies have explored *plag1* loss‐of‐function in this system. To address this, we employed antisense morpholino oligonucleotide (MO)‐mediated knockdown of *plag1* during early zebrafish embryogenesis to investigate its role in craniofacial patterning, neural development and behavioural outputs.

Quantitative phenotyping included high‐resolution imaging of neuronal populations using transgenic lines, skeletal analysis of craniofacial cartilage structures and behavioural assays designed to probe locomotor activity and stimulus‐evoked responses. Our behavioural screen was motivated by early observations of abnormal swimming patterns in *plag1* morphants. To dissect this further, we employed two established paradigms: the light–dark challenge, which quantifies anxiety‐like behaviour through changes in photic‐driven activity (Rock et al. 2022; Tuz‐Sasik et al. 2022), and the startle response assay, which measures sensorimotor function and habituation to acoustic stimuli (Beppi et al. 2021).

Although PLAG1 has been implicated in growth restriction and craniofacial anomalies in human SRS, its contribution to neurobehavioural development remains poorly understood. PLAG1 regulates conserved developmental pathways involved in craniofacial patterning and neural development, suggesting that early behavioural alterations in zebrafish may reflect disruptions in phenotypic domains that are clinically relevant in SRS. Our study therefore provides an initial vertebrate framework for examining how plag1 influences early behavioural and morphological development. We hypothesise that *plag1* is required for proper craniofacial development and contributes to the regulation of sensorimotor behaviours during early neurodevelopment, and therefore the zebrafish are likely to present a compelling model of SRS. This study represents the first functional interrogation of *plag1* in any vertebrate model with respect to neurodevelopmental and behavioural outcomes.

## 2 Materials and Methods

### 2.1 Zebrafish Husbandry and Embryo Collection

All zebrafish ( *D. rerio* ) lines were maintained under standard conditions at La Trobe University in accordance with institutional animal ethics approval (AEC16‐091, AEC23‐011). The strains used included wild‐type Tübingen (WT), *Tg (elavl3:EGFP*; in which GFP is expressed throughout the entire brain (Vandestadt et al. 2021) and *Tg (mnx1:BFP*; where BFP is expressed within the spinal cord and motor neurons (Vandestadt et al. 2021), kindly provided by Dr. Samuel Crossman and A/Prof. Jan Kaslin, Monash University. Adult fish (3 months old) were housed in Tecniplast recirculating tanks and bred under conditions designed to mimic natural spawning. Males and females were separated overnight by a transparent divider. After a 10‐h dark period, lights were switched on to induce spawning, at which point the divider was removed. Fertilised embryos were collected within 30 min, washed in E3 medium (5 mM NaCl, 0.17 mM KCl, 0.33 mM CaCl_2_ and 0.33 mM MgSO₄) and incubated at 28°C.

### 2.2 Morpholino Oligonucleotides and Zebrafish Micro‐Injection

A translation‐blocking morpholino (morpholino oligonucleotides [MO]) targeting the ATG start site of *plag1* mRNA was synthesised by Gene Tools LLC (5′‐TTCTCATATTTTTTGCTGGACGCGG‐3′). A standard control morpholino (5′‐CCTCTTACCTCAGTTACAATTTATA‐3′) served as the negative control. Dose–response experiments established that 500 μM was the optimal concentration for robust translational inhibition with minimal off‐target effects. MOs were injected at the one‐ to two‐cell stage into the yolk in a volume of ~2 nL using a pneumatic microinjection apparatus. Injected embryos were raised in E3 medium at 28°C and staged according to (Kimmel et al. 1995).

### 2.3 CRISPR‐Cas9 Targeting of *plag1*

Four CRISPR RNAs (crRNAs) targeting exon 3 of *plag1*, the largest exon conserved between the *plag1‐201* and *plag1‐202* transcripts, were designed using the Integrated DNA Technologies (IDT) guide RNA design tool. Guides were selected for low predicted off‐target activity and > 70% predicted on‐target efficiency and synthesised by IDT (USA). The crRNA sequences were: Guide 1, 5′‐CCATACACACGACCCCAACAGTTTTAGAGCTATGCT‐3′; Guide 2, 5′‐GTGTATGGAGATGGTTCTTTGTTTTAGAGCTATGCT‐3′; Guide 3, 5′‐TGTTGCTGGAGCACCTACGTGTTTTAGAGCTATGCT‐3′; and Guide 4, 5′‐TGGGATTCCATCAAATCTACGTTTTAGAGCTATGCT‐3′. For guide RNA preparation, 300 pmol of each crRNA was separately combined with 300 pmol trans‐activating CRISPR RNA (tracrRNA) in nuclease‐free duplex buffer (IDT) and incubated at 95°C for 5 min. The four resulting guide RNA complexes were combined in an injection mixture at a final concentration of 172 ng/μL each, together with 700 ng/μL Cas9 HiFi v3 protein (IDT), 250 mM KCl and 0.008% phenol red. Control embryos received vehicle containing 250 mM KCl and 0.008% phenol red without guide RNAs or Cas9, controlling for effects associated with microinjection. Injection mixtures were briefly centrifuged and maintained on ice before use. Mixtures were microinjected directly into one‐cell‐stage zebrafish embryos, which were subsequently maintained at 28°C.

### 2.4 Guide Validation

CRISPR–Cas9‐mediated editing of *plag1* was assessed in six guide‐injected and six control embryos collected at 24‐h postfertilisation (hpf). Genomic regions encompassing the four crRNA target sites were amplified using three primer pairs, with Guides 1 and 2 assessed using a single primer pair due to their overlapping target regions. Primer sequences were: Guide 1/2, F 5′‐GTATAAACTCCTACGGCACATGG‐3′ and R 5′‐TCGTGTTGTAACTCTTGTCGCA‐3′; Guide 3, F 5′‐AGCGTGGAGACCTCACCTGC‐3′ and R 5′‐TGGCGTCGCACATCCTTACG‐3′; and Guide 4, F 5′‐CTGCGCCCAACGATTCGGTC‐3′ and R 5′‐GGCGGTGGTGAGTAGGAGGC‐3′.

Genomic DNA was extracted using a modified HotSHOT method. Individual embryos were lysed in 30 μL 50 mM NaOH at 95°C for 25 min, cooled at 4°C for 5 min and neutralised with 3 μL 1 M Tris–HCl. Samples were briefly vortexed and centrifuged, and the supernatant was used as genomic DNA template. Target regions were amplified using GoTaq Green Master Mix (Promega) according to the manufacturer's instructions.

### 2.5 Preparation of Live Embryos and Larvae for Imaging

To inhibit pigmentation, embryos were incubated from 24 hpf in E3 medium supplemented with 0.3% 1‐phenyl‐2‐thiourea (PTU). Prior to imaging, larvae were anaesthetised in tricaine (MS‐222; 500 μL of 50× stock per 25 mL E3). Anaesthetised larvae (5–10 per experiment) were transferred to a 35‐mm MatTek glass‐bottom dish. After removal of excess medium, embryos were immobilised in 1% low‐melting‐point agarose in anaesthetic medium. Larvae were oriented using an eyelash tool and allowed to set for ~5 min before imaging.

### 2.6 Qualitative Behavioural Analysis

At 5 dpf, the touch‐evoked escape response was evaluated by gently stimulating the head region with a fine pipette tip. Behavioural outcomes were scored manually by blinded observers as performing an ‘immediate,’ ‘delayed’ or ‘absent’ response. Concurrently, locomotor output was classified as ‘standard,’ ‘subtle’ or ‘no movement.’ For clarity, ‘immediate’ was defined as initiation of movement within 1 s of head stimulation; ‘delayed’ as initiation between 1–5 s; and ‘absent’ as no movement within 5 s. Locomotor output was further classified as ‘standard’ (robust escape swim covering > 5 mm in a single bout), ‘subtle’ (short displacement ≤ 5 mm, typically one or two tail flicks without sustained swimming) or ‘no movement’ (no detectable displacement). This simple yet robust approach allowed for the evaluation of sensory and motor function in the zebrafish larvae.

### 2.7 Quantitative Analysis of Brain Morphology

To assess brain development, *Tg (elavl3:EGFP)* embryos were injected with either *plag1* MO or control MO and collected at 24, 48 and 72 hpf. Larvae were prepared for imaging as described above. Confocal images were acquired using a Zeiss LSM 780 or Nikon TiE inverted fluorescence microscope. GFP signal permitted visualisation of neuronal architecture, with anatomical boundaries delineated based on fluorescence intensity and established landmarks. Image analysis was performed using ImageJ (NIH) and NIS Elements v3.22. Brain regional area (μm^2^) was measured for forebrain, midbrain and hindbrain from five to seven larvae per experimental condition.

### 2.8 Quantitative Analysis of Motor Neuron Development

Motor neuron morphology was assessed in *Tg (mnx1:BFP)* embryos injected with *plag1* or control MOs. Embryos were fixed and imaged at 72 hpf using an Andor Dragonfly 202 spinning‐disk confocal microscope. Caudal primary (CaP) motor neuron axons (*n* = 10 CaP neurons per larva) were manually traced, and axonal length quantified using Imaris v9.5 software (Bitplane). Primary branch points were counted manually. Data were derived from five to seven larvae at 48 hpf and six to eight larvae at 72 hpf per group.

### 2.9 Automated Behavioural Phenotyping

For all behavioural assays, 5 dpf larvae were tested in 48‐well plates, with one larva per well in 1 mL E3 medium. Animals were acclimated for ≥ 30 min before testing. All experiments were performed in the DanioVision Observation Chamber (Noldus), with environmental temperature maintained at 28°C and data captured at 30 fps using EthoVision XT software (Noldus). For analysis of thigmotaxis, each well had a diameter of 11.4 mm and was divided into centre and edge zones. The centre zone was defined as a circular region 6.9 mm in diameter, with the remaining peripheral region defined as the edge. The percentage of time spent within each zone was quantified using EthoVision XT.

### 2.10 Spontaneous Locomotion Assay

Larvae were assessed between 9:00 and 12:00, a period of maximal baseline activity (MacPhail et al. 2009). After a 10‐min acclimation, locomotor behaviour was recorded for 10 min under infrared illumination. Each larva was recorded for 10 min. Parameters analysed included total distance moved (mm), mean swimming velocity (mm/s) and spatial occupancy (central vs. peripheral well zones).

### 2.11 Dark–Light–Dark Transition Assay

Photic sensitivity and anxiety‐like behaviour were assessed at 5 dpf using a dark–light–dark (DLD) paradigm adapted from (van den Bos et al. 2017). Testing was conducted between 13:00 and 18:00, when larval activity is most stable (MacPhail et al. 2009). Individual larvae were placed in 48‐well plates (1 mL E3/well), acclimated for 20 min under ambient light (500–650 lx), then exposed to a lighting sequence of 10 min dark, 10 min bright illumination (~3000 lx) and 10 min dark, at 28°C in the DanioVision Observation Chamber. Behaviour was recorded at 30 fps (EthoVision XT) and analysed across three domains: baseline locomotion, response to light onset and response to light offset. Activity was quantified as total distance moved (mm) per phase. Transition‐specific responses were measured from 30 s epochs: (i) change in maximum velocity (Δ*V*_max_), defined as the difference between peak velocity in the new and preceding phase and (ii) change in distance travelled (Δ*D*), calculated as Dlight − Ddark across the dark–light transition. Light‐offset responses were similarly assessed by Δ*V*_max_ between the initial dark period and the preceding light phase. These parameters provided a sensitive readout of locomotor reactivity and anxiety‐like behaviour in *plag1*‐morphants.

### 2.12 Startle Response Assay

To assess sensorimotor reactivity and habituation, 5 dpf larvae were tested in an acoustic startle paradigm immediately after the DLD assay. Larvae remained in their original 48‐well plates and acclimated for 10 min in the DanioVision chamber (Noldus). Two stimulation phases were delivered at intensity 6/8: (i) 10 discrete stimuli with 20‐s interstimulus intervals (ISIs) to assess baseline startle and habituation, followed by (ii) a 10‐min rest and then 30 rapid stimuli at 1s ISI to probe responses under sustained stimulation. Behaviour was recorded at 30 fps (EthoVision XT), and maximum velocity (mm/s) after each stimulus was quantified as the primary readout. Larvae failing to respond to the initial stimulus were excluded to control for morpholino‐injection–related defects.

## 3 Results

### 3.1 Knockdown of plag1 Leads to Minor Phenotypic Abnormalities in the Craniofacial Skeleton, but Not Body Length or Brain Development

We have previously demonstrated that deletion of *Plag1* in mouse results in a significant growth‐restriction phenotype, with *Plag1* ^*−/−*^ embryos at embryonic day 14.5 exhibiting an average 13% reduction in body weight compared to their wild‐type littermates (Gasperoni et al. 2024). To this end, body length was measured in both control and *plag1* morpholino‐injected larvae at 5‐day postfertilisation (dpf; Figure 1A–C). A minimum of 10 larvae per group were assessed, with measurements taken from the tip of the snout to the end of the caudal fin. Control‐injected larvae exhibited a mean body length of 4.00 ± 0.03 mm, while *plag1* morphants measured 3.90 ± 0.05 mm (mean ± SEM), indicating no significant alteration to body length in zebrafish. These data suggest that, unlike in the mouse, *plag1* knockdown does not markedly impact early zebrafish somatic growth.

**FIGURE 1:**
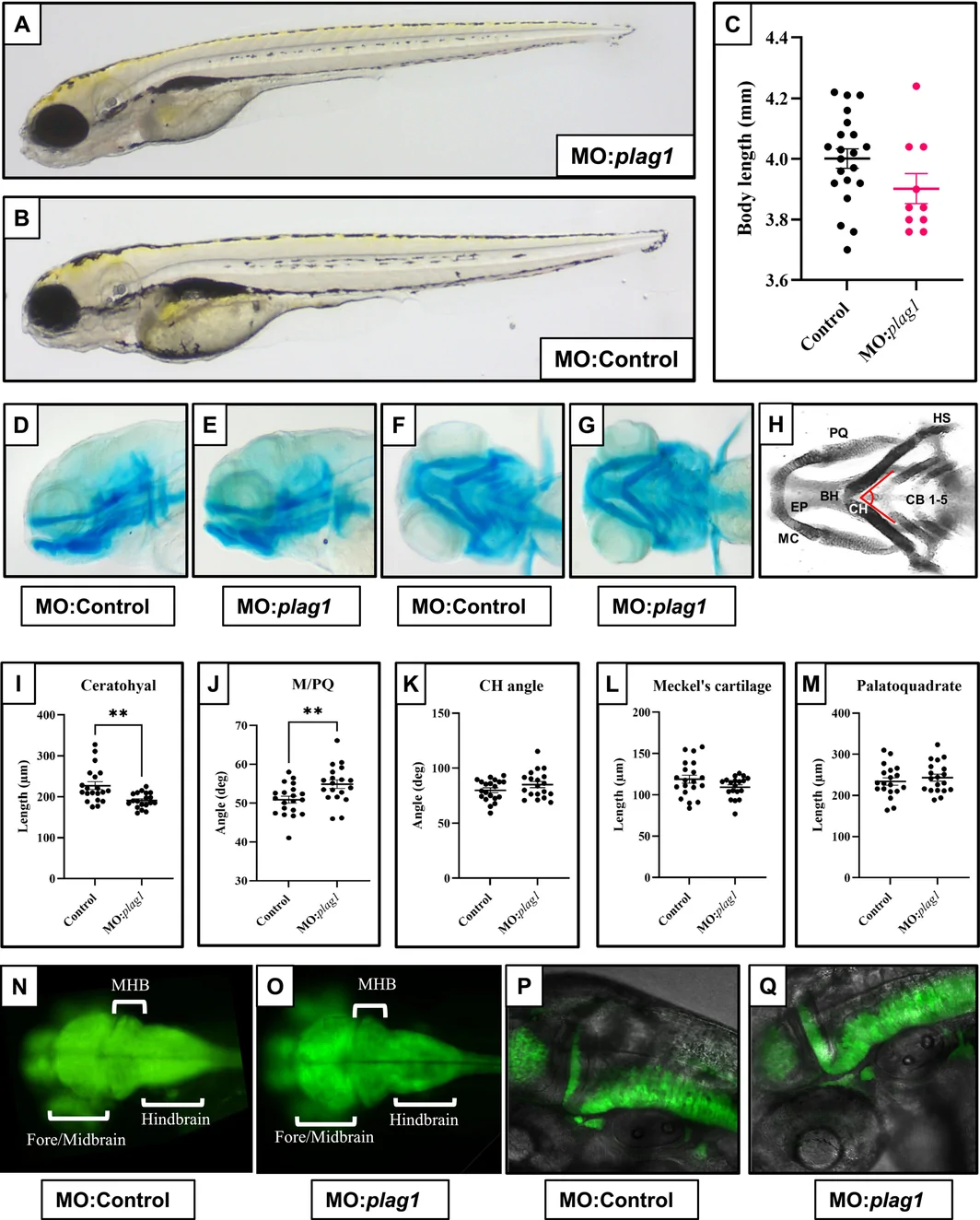
Plag1 knockdown does not alter gross morphology or overall brain size but disrupts craniofacial patterning. Representative brightfield images of MO:plag1 (A) and control (B) larvae at 5‐day postfertilisation (dpf). Quantification of body length (mm) (C), measured from the anterior tip of the head to the tip of the tail (*n* ≥ 10). Lateral views of Alcian blue‐stained craniofacial structures in control (D) and MO:plag1 larvae (E), and ventral views of the same in control (F) and MO:plag1 (G). Schematic diagram showing the positioning of Meckels cartilage (MC), ethmoid plate (EP). Basihyal (BH), palatoquadrate (PQ), hyosymplectic (HS) and ceratobranchial 1–5 (CB 1–5) craniofacial cartilages, alongside the angle of the ceratohyal, shown in red (H). Quantification of ceratohyal cartilage length (μm) (I), Meckels–palatoquadrate (M/PQ) angle (J), ceratohyal angle (K), Meckels cartilage length (L) and palatoquadrate length (M), (*n* ≥ 10). Representative confocal images of neural architecture using *Tg* (*elavl3:EGFP*) at 24 hpf (N,O) and 48 hpf (P,Q) in control and MO:plag1 larvae, respectively (*n* = 3 per group). *p ≤ 0.05 (unpaired *t*‐test). Error bars represent standard error of the mean (SEM).

Given the craniofacial abnormalities associated with *PLAG1* dysregulation in SRS, we investigated whether *plag1* knockdown in zebrafish produced analogous defects in craniofacial cartilage development (Figure 1D–M). At 4‐day postfertilisation (dpf), Alcian Blue staining was used to visualise craniofacial structures (Figure 1D–G), and morphometric analyses were conducted on lateral and ventral views of control and *plag1* morphant larvae (Figure 1H–M). Quantitative analyses revealed a significant reduction in ceratohyal (CH) cartilage length in *plag1* morphants (Figure 1I), as well as a marked increase in the Meckel's cartilage (MC)‐palatoquadrate (M/PQ) angle (Figure 1J), indicating altered positioning of mandibular elements. In contrast, the angle of the ceratohyal (Figure 1K), the lengths of Meckel's cartilage (Figure 1L) and the palatoquadrate (PQ; Figure 1M) were not significantly affected. These findings suggest a role for *plag1* in the spatial organisation of craniofacial structures, supporting a putative contribution to the subtle craniofacial phenotypes observed in SRS.

Lastly, to determine whether *plag1* knockdown impacts early brain development, we examined neural morphology at 24‐ and 48‐h postfertilisation (hpf) in *Tg (elavl3:EGFP)* embryos, a transgenic line in which postmitotic neurons are fluorescently labelled (Figure 1N–Q). This allowed visualisation and comparison of overall brain structure and neuronal architecture in both control and *plag1* morphant embryos. Representative confocal images at 24 hpf (Figure 1N,O) and 48 hpf (Figure 1P,Q) revealed no gross morphological abnormalities, with overall brain size, formation of the midbrain‐hindbrain boundary (MHB) and regional organisation largely preserved. Quantitation of forebrain, midbrain and hindbrain area at 72 hpf shows that the total brain area does not differ significantly between the two groups (control: 100 ± 1.14 μm^2^; MO:*plag1*: 98.1 ± 3.13 μm^2^; *p* = 0.2). Although no gross structural abnormalities were detected, our analyses were limited to overall morphology and did not assess finer‐scale regional volumes, neuronal density or organisation of specific nuclei. Therefore, the presence of more subtle circuit‐level alterations that fall below the resolution of our imaging pipeline cannot be excluded.

### 3.2 Plag1 Knockdown Induces Thigmotaxis and Altered Stimulus‐Evoked Motor Responses

During initial postinjection screening, qualitative observation of swimming behaviour in group‐housed larvae revealed that *plag1* morphants consistently exhibited thigmotaxis—defined as a strong preference for swimming along the periphery of the Petri dish or ‘wall‐hugging’ behaviour. This phenotype was not apparent in control‐injected siblings. To quantify this observation, the proportion of larvae occupying the outermost edge of the dish at 5 dpf was recorded, consistent with established methods for assessing thigmotactic behaviour (Schnörr et al. 2012). *Plag1* morphants exhibited a significantly higher incidence of thigmotaxis (45.5% ± 4.5%; *n* = 222) compared to controls (28.6% ± 3.0%; *n* = 284; *p* = 0.02), suggesting increased anxiety‐like behaviour and altered spatial preference in a social context (Figure 2A–C). To further assess sensorimotor function, we employed a touch‐evoked response assay at 5 dpf. The majority of larvae in both groups exhibited an immediate reaction to head stimulation (control: 88% ± 3.6%; *n* = 78; *plag1* morphants: 92% ± 2.0%; *n* = 189), indicating intact basic sensory detection (Figure 2D). However, assessment of the motor response revealed a striking difference in movement character. While nearly all control larvae (99% ± 1.3%) displayed robust escape swimming typical of the touch‐evoked response, the vast majority of *plag1* morphants (92% ± 2.0%) exhibited only subtle, short‐distance movements away from the stimulus (Figure 2E). This phenotype was consistent across embryos analysed from three independent clutches of embryos (*n* = 3 plates per clutch) and suggested a specific disruption to stimulus‐driven motor output. Together, these findings support a role for *plag1* in modulating early behavioural responses and locomotor coordination.

**FIGURE 2:**
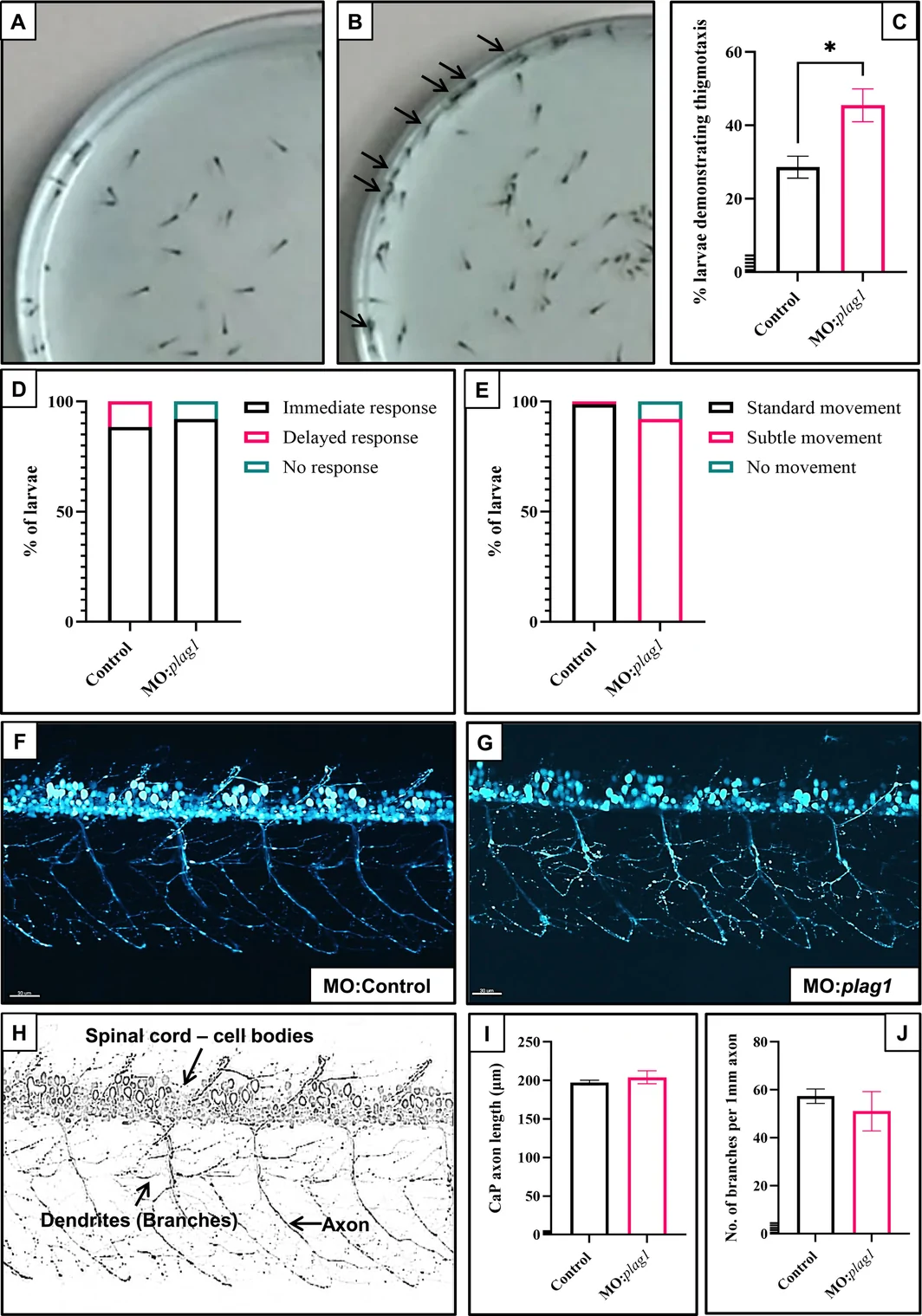
Plag1 knock‐down induces thigmotaxis and subtle changes in touch‐evoked responses but does not affect motor neuron development. Representative images of 5 dpf larvae housed in group conditions in Petri dishes: control (A) and MO:plag1 (B), from the same clutches (*n* = 60–90). Quantification of the proportion of larvae that displayed thigmotaxis (C). Percentage of larvae responding to touch stimulus (D), and assessment of movement type following touch stimulus (E). Confocal images of caudal primary (CaP) motor neurons in *Tg* (*mnx1:BFP*) fish at 72 hpf in control (F) and MO:plag1 (G) larvae. Schematic diagram (H) showing dorsal location of the spinal cord and neuronal cell bodies, alongside the ventrally descending axons and dendrites (branches). Quantification of CaP axon length (μm) (I) and number of primary branches per 1 mm of axon (J). Axon length and branching were quantified from a minimum of 10 embryos per group. Immediate = response within 1 s; delayed = 1–5 s; absent = no response. Standard = escape swim > 5 mm; subtle ≤ 5‐mm displacement. Scale bar = 30 μm (F–G). *p ≤ 0.05 (unpaired *t*‐test). Error bars represent SEM.

### 3.3 Plag1 Knockdown Does Not Impair CaP Motor Neuron Axon Growth or Branching

As a prelude to investigating functional motor outputs, we first assessed whether the altered swimming behaviours observed in *plag1* morphants could be attributed to underlying defects in motor neuron development. Specifically, we examined the morphology of caudal primary (CaP) motor neurons, which extend ventrally from the spinal cord and serve as key pioneers in establishing early neuromuscular connectivity (Sato‐Maeda et al. 2008). Analyses were conducted using the *Tg (mnx1:BFP)* transgenic line at 48‐ and 72‐h postfertilisation (hpf), a time window during which CaP neurons complete their initial trajectory and begin to arborise (Figure 2F–H). Quantification of axonal outgrowth from *n* ≥ 10 embryos per group at 72 hpf revealed no significant difference in axon length between control‐injected larvae (197.2 ± 3.1 μm; *n* = 12) and MO:*plag1*‐injected larvae (204.1 ± 8.4 μm; *n* = 5; Figure 2I), indicating that *plag1* knockdown does not impair CaP axon extension.

We next examined the arborisation of CaP neurons, as axonal branching is essential for effective target innervation and motor coordination. The number of branches per millimetre of axon was quantified at 72 hpf (Figure 2J) from *n* ≥ 10 embryos per group. No significant differences were detected between control (57.4 ± 3.0) and MO:*plag1* larvae (51.1 ± 8.2), suggesting that axonal complexity and innervation potential are preserved following down‐regulation of *plag1*. Together, these findings demonstrate that inhibition of *plag1* does not impair the developmental growth or branching of CaP motor neurons during early embryogenesis.

### 3.4 Plag1 Deficiency Affects Behaviour, but Not Gross Locomotion in 5 Dpf Zebrafish Larvae

To determine whether the altered behavioural phenotypes observed in *plag1* morphants reflected deficits in locomotor capacity or were indicative of behavioural choice, we conducted high‐resolution tracking of individual larvae in an isolated context (Figure 3A–E). Quantitative analysis revealed no significant differences in either metric between experimental groups. *Plag1* morphants travelled an average of 1322 ± 81.8 mm (*n* = 142) compared to 1467 ± 78.8 mm in controls (*n* = 136), while mean swimming velocities were similarly indistinguishable (morphants: 2.44 ± 0.14 mm/s; controls: 2.09 ± 0.08 mm/s; Figure 3C,D). However, analysis of spatial occupancy within the wells (Figure 3E) revealed a marked difference in behaviour. While total movement was equivalent, *plag1* morphants exhibited a significantly higher tendency to remain near the periphery of the well, resulting in reduced coverage of the central area, mirroring the thigmotactic behaviour seen in the larger petri dishes. Quantification of well occupancy confirmed this spatial preference was statistically significant (****p < 0.0001, Fisher's exact test; Figure 3E). These findings suggest that while *plag1* morphants retain normal locomotor capability, their altered swim trajectories reflect a shift in behavioural choices rather than impaired motor function.

**FIGURE 3:**
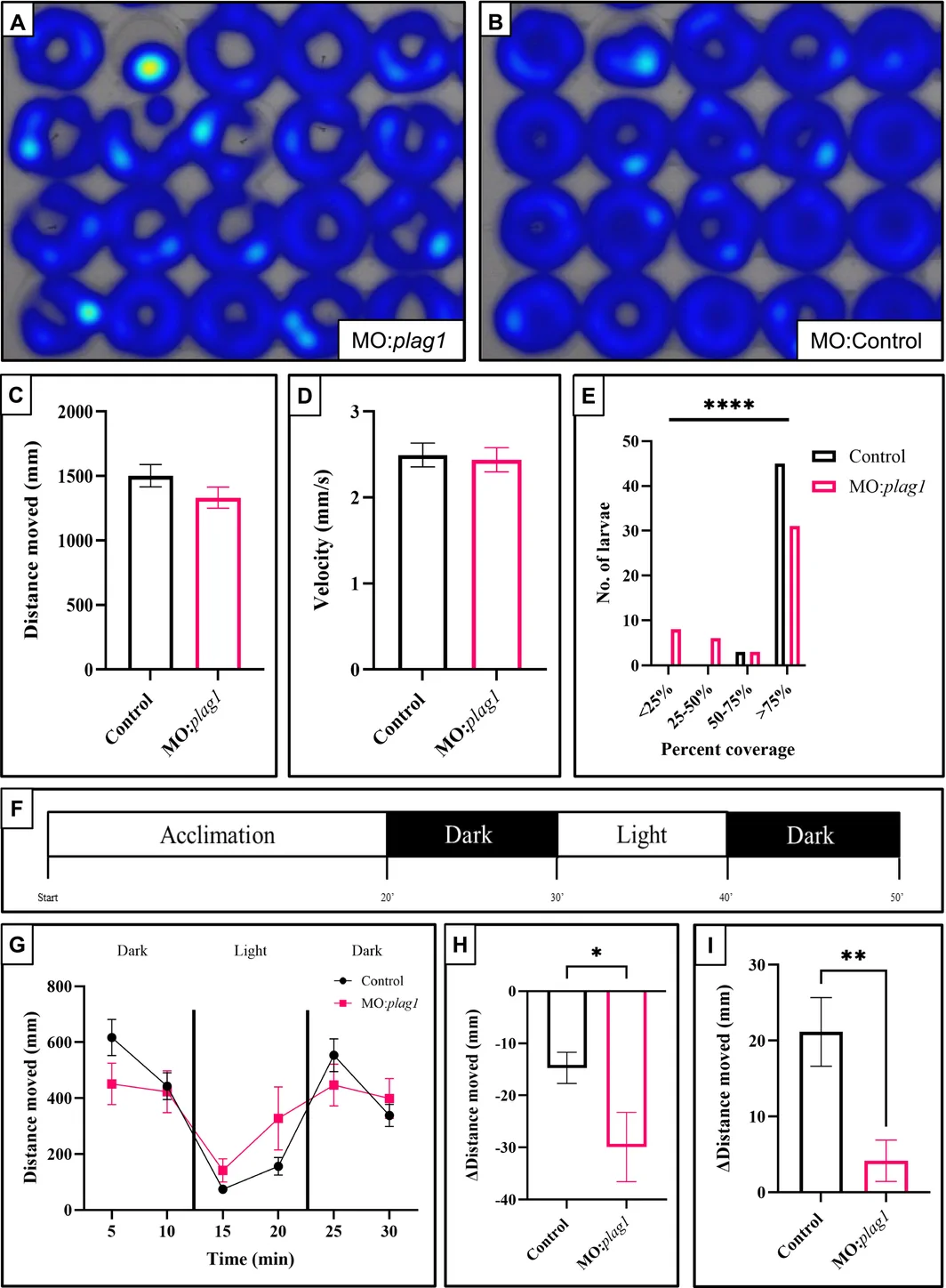
Locomotor capacity is preserved in isolated *plag1* morphants, but behavioural modulation is altered in light–dark transition assays. Representative DanioVision heatmaps of individual MO:plag1 (A) and control (B) larvae during the 10‐min free‐swimming locomotion assay (*n* ≥ 136). Quantification of total distance travelled (mm) (C) and mean swimming velocity (mm/s) (D) show no significant difference between control and MO:plag1 larvae. Percentage of well area occupied by larvae during the test, used as a proxy for thigmotaxis (E); ****p < 0.0001 (Fishers exact test). Schematic of dark–light–dark activity assay showing timing of light phases (F). Velocity over time during dark, light and dark periods (G). Change in swimming distance (Δ) in the first 30 s of light versus last 30 s of prior dark (dark‐to‐light transition), *p = 0.04 (Welchs *t*‐test) (H). Change in swimming distance (Δ) in the first 30 s of dark versus last 30 s of prior light (light‐to‐dark transition), **p = 0.002 (Welchs *t*‐test) (I). Error bars represent SEM. Mixed‐effects analysis confirmed a significant effect of time across the entire experiment (*F*[2.460, 205.7] = 35.35; *p* < 0.0001), although no overall difference was detected between groups (*F*[1,86] = 9.79) (*n* = 48).

### 3.5 Inhibition of Plag1 Results in Heightened Anxiety

This spatial preference, also observed in anxiety paradigms, is suggestive of an anxiety‐like behavioural phenotype in *plag1*‐deficient larvae. To investigate this swimming behaviour further, the light–dark paradigm assay was used to assess anxiety‐like responses by tracking changes in locomotion triggered by sudden illumination changes.

Control and *plag1* morphant larvae were tracked over the following three consecutive 10‐min phases: dark, light and dark (Figure 3F–G). Each phase was subdivided into two 5‐min blocks to evaluate intraphase dynamics. During the initial 10‐min dark phase, control larvae significantly reduced their swimming over time (5 min: 617.2 ± 52.9 mm; 10 min: 442.8 ± 37.9 mm; *p* < 0.0001), consistent with acclimation. In contrast, *plag1* morphants showed no such reduction (5 min: 450.9 ± 53.9 mm; 10 min: 422.8 ± 153.2 mm), suggesting an impaired habituation response to constant darkness. No significant difference was observed between the groups in overall distance swum during this phase.

Following the dark‐to‐light transition, both control and *plag1* larvae exhibited a significant reduction in distance swum (control: −14.71 ± 3.0 mm; *p* < 0.0001; MO:plag1: −29.89 ± 6.64 mm; *p* < 0.0001), indicative of an acute photic response. However, the reduction was significantly greater in *plag1* morphants (*p* = 0.04; Figure 3H), suggesting heightened sensitivity or altered modulation of behaviour in response to illumination.

Interestingly, while both groups initially displayed reduced activity in the light, they subsequently exhibited increased locomotion as the light period progressed. Control larvae increased their distance travelled from 74.6 ± 13.7 mm (15 min) to 156.5 ± 28.0 mm (20 min; *p* = 0.04), and *plag1* morphants showed an even greater increase (141.4 ± 47.7 mm to 327.5 ± 73.4 mm; *p* < 0.0001), indicating that *plag1* deficiency does not impair the capacity to respond to prolonged light exposure. However, during the subsequent light‐to‐dark transition, control larvae demonstrated a robust increase in movement (21.16 ± 4.53 mm; *p* < 0.0001), whereas *plag1* morphants did not show a significant change (4.16 ± 2.72 mm), and this blunted response was significantly different from controls (*p* = 0.002; Figure 3I). This suggests that *plag1* morphants are less responsive to sudden darkness, in contrast to their heightened reaction to light onset.

Finally, during the second dark phase, control larvae again showed a typical reduction in locomotion over time (25 min: 533.4 ± 48.7 mm; 30 min: 338.5 ± 36.9 mm; *p* < 0.0001), indicative of behavioural acclimation. No such decline was observed in *plag1* morphants (25 min: 446.7 ± 58.9 mm; 30 min: 398.8 ± 50.9 mm), which maintained consistent levels of activity, similar to those recorded during the initial dark phase (Figure 3I).

Together, these data demonstrate that plag1‐deficient larvae exhibit heightened sensitivity to light onset and reduced responsiveness to light offset, alongside impaired behavioural habituation in darkness, features consistent with altered sensory‐driven behavioural modulation, which may include, but are not limited to anxiety‐like processes. This is reminiscent of aspects of the anxiety‐like traits and altered sensory processing observed in SRS.

### 3.6 Plag1 Morphants Display Delayed Habituation and Heightened Responses to Acoustic Stimuli

We next sought to determine whether the heightened anxiety‐like behaviours in *plag1* morphants extended to other stress‐inducing paradigms. To this end, we employed a two‐phase acoustic startle and habituation assay designed to probe sensorimotor responsiveness, nonassociative learning and anxiety‐like reactivity (Figure 4A). The first phase consisted of 10 vibrational/acoustic stimuli delivered at 20‐s ISIs, spanning a total duration of 200 s. This protocol assesses the acute startle response and the capacity for habituation, which is a fundamental form of nonassociative learning. The second phase involved a rapid series of 30 stimuli at 1‐s ISI, designed to test anxiety‐like behaviour under sustained sensory stimulation, as such conditions have been shown to elicit heightened vigilance and stress‐related behaviours in larval zebrafish (Tan et al. 2022).

**FIGURE 4:**
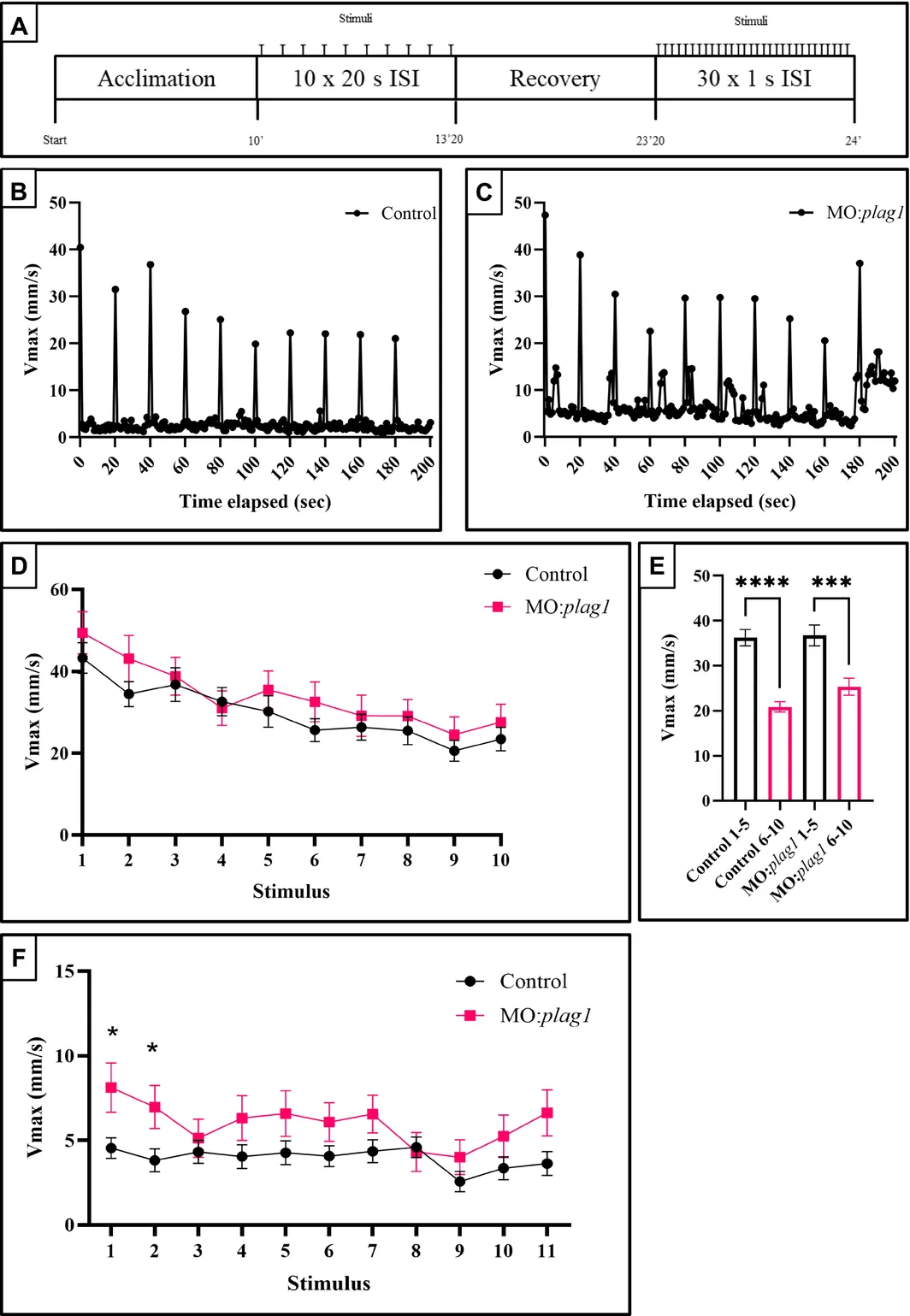
Plag1 morphants show delayed habituation to acoustic stimuli, indicating altered sensorimotor adaptation. Schematic of startle response and habituation assay timeline (A). Maximum velocity (*V*_max_; mm/s) of control (B) and MO:plag1 (C) larvae across 10 acoustic stimuli with 20‐s interstimulus intervals (ISI). Comparison of *V*_max_ between groups across all 10 stimuli (D). Mean *V*_max_ for early (stimuli 1–5) versus late (stimuli 6–10) stimuli for both groups, ***p < 0.001; ****p < 0.0001 (two‐way ANOVA) (E). ‘Resting’ velocity (*V*_max_) in the 5 s prior to each stimulus across groups, *p ≤ 0.05 (Tukey's multiple comparisons test) (F). Error bars represent SEM (*n* = 48).

Control larvae showed significant habituation from the sixth stimulus onward, with progressively lower peak velocities compared to the first stimulus (Figure 4B–D; *p* = 0.02 to < 0.0001 across stimuli 6–10). In contrast, *plag1* morphants exhibited a more limited habituation profile: Only the ninth stimulus elicited a response significantly lower than that of the first (stimulus 1: 55.42 mm/s; stimulus 9: 29.62 mm/s; *p* = 0.04; Figure 4C,D), indicating a mild but measurable delay in habituation.

To further quantify this trend, average responses from the first five stimuli (stimuli 1–5) were compared to those from the latter five (stimuli 6–10; Figure 4E). Both groups exhibited reduced responses in the second half of the assay, but the magnitude of change differed. *Plag1* morphants showed a significant decrease in velocity from 36.35 ± 4.02 mm/s to 24.78 ± 3.57 mm/s (*p* = 0.0001), while controls showed a more pronounced decline (36.26 ± 1.80 mm/s to 21.15 ± 2.00 mm/s). These results suggest that although both groups can habituate to spaced stimuli, *plag1* morphants do so less efficiently, reflecting subtle disruptions in sensorimotor processing or central regulation of startle responsiveness.

In the second, rapid‐stimulus phase, controls displayed a steep and consistent decline in response following the initial startle (stimulus 1: 44.07 mm/s), with significantly reduced velocities from stimulus 2 onwards (*p* = 0.0073–0.0001). *Plag1* morphants also showed reduced responsiveness from stimulus 5 onwards (*p* = 0.0322–0.0001), suggesting a blunted recovery and an anxiety‐like behavioural profile consistent with their performance in the light–dark assay (Figure 4F).

Taken together, these findings demonstrate that *plag1* morphants exhibit a mild delay in habituation to spaced acoustic stimuli, and a heightened sensitivity to rapid, repeated stimuli. These phenotypes are consistent with increased baseline anxiety or altered regulation of sensorimotor gating, further implicating *plag1* in the modulation of behavioural reactivity and nonassociative learning. These findings identify behavioural consequences of *plag1* knockdown, although these do not begin to yet uncover the molecular or cellular pathways through which *plag1* exerts these effects. As Plag1 is a transcription factor, alterations likely arise from downstream changes in gene regulatory networks that remain to be characterised.

### 3.7 CRISPR/Cas9 Disruption of *plag1* Recapitulates Key Behavioural Phenotypes Observed Following Morpholino Knockdown

To independently validate the behavioural phenotypes identified following morpholino‐mediated *plag1* knockdown, we generated F0 *plag1* crispants using four guide RNAs targeting the *plag1* locus, with CRISPR/Cas9‐mediated editing confirmed by altered PCR product profiles consistent with mosaic indel formation (Figure S1A–D). Similar to *plag1* morphants, crispant larvae displayed a pronounced thigmotactic phenotype under group‐housed conditions, with 45.8% ± 4.8% of *plag1* crispants exhibiting peripheral localisation compared with 22.2% ± 4.6% of controls (*p* = 0.001; Figure 5A–C). This phenotype was also evident during individual tracking, where crispants spent significantly more time at the edge of the well than controls (78.3% ± 2.2% vs. 70.7% ± 2.1%; *p* = 0.0307; Figure 5D), with a corresponding trend towards reduced centre occupancy (21.2% ± 2.2% vs. 28.0% ± 2.0%; *p* = 0.06). In contrast to morphants, however, *plag1* crispants showed a modest reduction in overall locomotor activity, travelling a shorter total distance (1666 vs. 2233 mm; *p* = 0.01) and exhibiting a lower mean swimming velocity (2.802 vs. 3.785 mm/s; *p* = 0.01; Figure 5E,F). Responses to repeated acoustic stimulation demonstrated significant habituation across early and late stimulus blocks in both control and *plag1* crispant larvae (controls, *p* < 0.0001; crispants, *p* = 0.0005; Figure 5G,H), without the delayed habituation phenotype observed in morphants. Similarly, light–dark transition responses were largely preserved in crispants, although a nonsignificant trend towards a reduced dark‐to‐light response was observed (*p* = 0.07), while light‐to‐dark responses were indistinguishable from controls (*p* = 0.98; Figure S2A–C). Together, these data independently reproduce the robust thigmotactic phenotype associated with *plag1* deficiency, while revealing more variable effects on locomotor and stimulus‐dependent behaviours across the two transient loss‐of‐function approaches.

**FIGURE 5:**
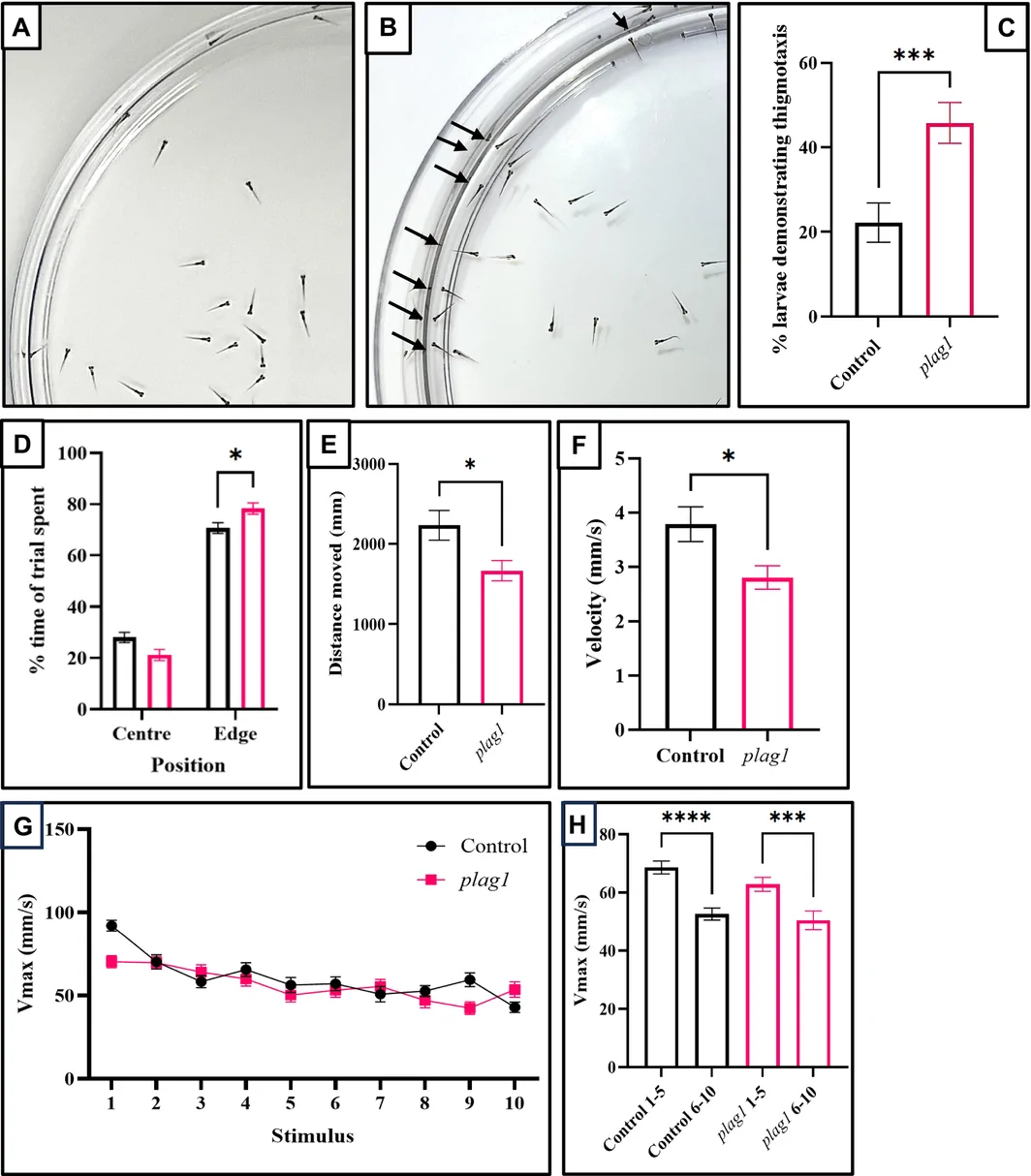
Plag1 crispants recapitulate altered thigmotaxis, locomotor activity and sensorimotor habituation phenotypes. Representative images of control (A) and *plag1* crispant (B) larvae displaying spatial distribution under group‐housed conditions. Quantification of the proportion of larvae displaying thigmotaxis (C), with increased thigmotaxis in *plag1* crispants (45.8% ± 4.8%) compared with controls (22.2% ± 4.6%); ***p = 0.001 (unpaired *t*‐test). Quantification of spatial preference in individually housed larvae, expressed as percentage of time spent in the centre or edge of the well (D). *plag1* crispants spent 21.2% ± 2.2% of time in the centre and 78.3% ± 2.2% at the edge, compared with 28.0% ± 2.0% and 70.7% ± 2.1%, respectively, for controls. The difference in time spent at the edge was significant (*p = 0.0307), whereas the difference in time spent in the centre did not reach statistical significance (*p* = 0.06; two‐way ANOVA with adjusted multiple comparisons; control *n* = 35, *plag1* crispant *n* = 47). Larvae for which tracking was unsuccessful for ≥ 5% of the recording period were excluded from analysis. Quantification of total distance travelled (mm) (E) and mean swimming velocity (mm/s) (F) showed reduced locomotor activity in *plag1* crispants compared with controls. Mean distance travelled was 1666 mm in *plag1* crispants and 2233 mm in controls (*p = 0.01, Welchs *t*‐test), while mean velocity was 2.802 mm/s and 3.785 mm/s, respectively (*p = 0.01, Welchs *t*‐test). Maximum velocity (*V*_max_; mm/s) during the acoustic startle habituation assay (G), with comparison of mean *V*_max_ for early (stimuli 1–5) and late (stimuli 6–10) stimuli (H). *V*_max_ decreased significantly between early and late stimuli in both controls (****p < 0.0001) and *plag1* crispants (***p = 0.0005; two‐way ANOVA with adjusted multiple comparisons). Error bars represent SEM.

## 4 Discussion

This study presents the first functional investigation of *plag1* in zebrafish, providing novel insights into its role in early neurobehavioural development. While *plag1* disruption did not impact gross morphology, somatic growth or motor neuron structure, it produced striking alterations in behavioural responses, particularly in paradigms associated with anxiety‐like phenotypes. These findings suggest that *plag1* plays a subtle but functionally significant role in modulating stimulus‐driven behaviour during early vertebrate development.

Consistent with previous findings in *Plag1*‐deficient mice, where growth restriction is a robust phenotype (Gasperoni et al. 2024; Hensen et al. 2004), we initially hypothesised that perturbation of plag1 function in zebrafish might similarly impair somatic development. However, transient disruption using morpholinos did not alter larval body length, suggesting that *plag1* may not be essential for axial elongation in zebrafish. This may reflect species‐specific differences in gene function, compensation by other transcriptional regulators or the partial nature of morpholino‐mediated inhibition. Notably, however, this phenotype aligns with observations in multiple congenital disorders, such as SRS, where growth abnormalities are typically variable and subtle in early development.

Expression of *plag1* in proliferative zones of the embryonic zebrafish brain and pharyngeal arches (including the ceratohyal region) has been documented (Pendeville et al. 2006), providing groundwork for our craniofacial and brain structure analyses and neurobehavioural assays. In terms of neural development, the overall structure and size of the zebrafish brain appeared grossly preserved, with no significant changes in total brain area between control and *plag1* morphants. Future studies employing neuronal density quantification and expression profiling of key patterning genes (e.g., *wnt1*, *eng2a* and *fgf8*) could reveal more nuanced roles for *plag1* in regional brain specification. It is noteworthy that while *Plag1* impacts neurogenesis, the related family member Plag‐like 2 (*Plagl2*) plays a complementary role in telencephalic patterning, highlighting the specificity and partial redundancy in this gene family (Adnani et al. 2018).

Craniofacial analysis, by contrast, revealed significant and reproducible abnormalities in *plag1* morphants, including reduced ceratohyal length and altered Meckel's/Palatoquadrate angle. While the specific craniofacial elements differ across species, the craniofacial changes observed following morpholino‐mediated *plag1* perturbation are consistent with disrupted pharyngeal arch patterning, a developmental domain also affected in SRS. Thus, we consider the zebrafish model to partially recapitulate the developmental directionality—albeit not exact morphology—of human craniofacial involvement.

The most striking findings of this study emerged from the behavioural assays. Previously, the role of Plag1 in behaviour had been examined in adult mice, where our prior foundational behavioural study revealed that PLAG1‐KO mice exhibit a mild phenotype; their overall circadian activity, locomotion, sociability, memory and cognitive functions are largely unaltered, although adult mice did show distinct deficits in specific sensorimotor functions, namely fear conditioning (cued), prepulse inhibition of the startle reflex and Preyer's reflex, suggesting some anxiety‐like behaviours and potential auditory circuit dysfunction (Tran et al. 2021). These findings prompted behavioural analysis of our zebrafish morphants. Initially, larvae following morpholino‐mediated *plag1* perturbation displayed prominent thigmotaxis when housed in groups—a wall‐hugging behaviour commonly associated with anxiety‐like states. This social sensitivity may point towards altered processing of environmental cues, potentially modelling aspects of social anxiety or vigilance, features commonly reported in SRS despite intact motor capabilities.

To probe this phenotype further, we employed two well‐established behavioural assays. In the light–dark transition assay, *plag1* morphants exhibited blunted responses to both light‐on and light‐off transitions, reflecting a dysregulated response to environmental change. While both groups ultimately adapted to prolonged illumination, *plag1* morphants failed to reduce activity in darkness, further supporting a disruption in behavioural modulation rather than locomotor capacity. The acoustic startle assay offered additional mechanistic insight. In the intermediate ISI paradigm, *plag1* morphants showed a mild delay in habituation, responding robustly to early stimuli but failing to adapt efficiently over time. Interestingly, their responses in the rapid ISI paradigm were broadly comparable to controls, suggesting that the deficit is context‐dependent and not a result of generalised hyperreactivity. Delayed habituation has been linked to deficits in sensorimotor gating and is a common feature of several neurodevelopmental disorders, including those characterised by increased anxiety or altered learning dynamics.

Importantly, the increased thigmotaxis observed following morpholino‐mediated *plag1* knockdown was independently reproduced in F0 *plag1* crispants, strengthening the conclusion that altered spatial preference is a genuine consequence of perturbed *plag1* function rather than a morpholino‐specific effect. However, the behavioural phenotypes were not identical between the two models. Whereas morphants retained normal gross locomotor capacity but showed altered responses to light–dark transitions and delayed acoustic habituation, crispants exhibited a modest reduction in basal locomotor activity while showing comparatively preserved responses across both sensory paradigms. Such differences are consistent with the fact that morpholino perturbation and CRISPR‐based gene disruption are complementary, yet biologically distinct, approaches. Morpholinos produce an acute, titratable and transient disruption of gene function during early development, making them well suited to investigating dosage‐sensitive and early developmental roles while offering limited opportunity for the adaptive or compensatory responses that may accompany genomic mutation. By contrast, CRISPR‐based disruption alters the genomic locus directly and, in stable mutant lines, can generate heritable loss of function suitable for analysis across development. F0 crispants, however, are inherently mosaic, such that individual larvae may carry different combinations and proportions of edited and unedited alleles across tissues. Consequently, the magnitude, spatial distribution and developmental timing of *plag1* disruption are unlikely to be equivalent between morphants and crispants. Mosaicism, variable editing efficiency, residual *plag1* activity and potential transcriptional adaptation may therefore contribute to differences in phenotype penetrance and severity. Nevertheless, convergence of both approaches on increased thigmotaxis identifies altered spatial preference as the most reproducible behavioural consequence of early *plag1* disruption, whereas differences in locomotor and stimulus‐evoked phenotypes suggest that these subtler behavioural outputs may be particularly sensitive to the extent and timing of *plag1* perturbation. Such divergence between morpholino and genetic models is well documented in zebrafish; for example, *egfl7* morphants display severe vascular defects that are largely absent in *egfl7* mutants (Rossi et al. 2015; El‐Brolosy and Stainier 2017), with similar discrepancies reported for *klf2a* (Novodvorsky et al. 2015) and *atoh8* (Place and Smith 2017). Together, these observations highlight that morphant, crispant and stable mutant approaches can reveal overlapping but nonidentical aspects of gene function, and support the complementary use of multiple strategies when interpreting developmental phenotypes.

To fully evaluate the contribution of *plag1* to complex behaviours at juvenile and adult stages—particularly those involving social interaction, memory or sustained anxiety—our current work is focussed on generating stable *plag1* mutant zebrafish lines. A limitation of this study is that the efficiency of the *plag1* morpholino was not directly validated at the transcript or protein level. While the concordant thigmotaxis phenotype in independent F0 crispants supports a gene‐specific effect, these experiments do not quantitate the extent of morpholino‐mediated knockdown. Future studies using stable, genetically validated *plag1* mutant lines will help clarify the effects of residual gene activity and developmental compensation. Such models would permit longitudinal studies and allow exploration of whether early behavioural disruptions persist or evolve as development proceeds. Additionally, as a transcription factor, Plag1 is likely to drive the expression of multiple target genes; uncovering conserved genetic networks will also be made possible through the future generation of Plag‐1 CRISPR deletion models.

Although gross brain architecture appears intact, the behavioural phenotypes imply circuit‐level neural connectivity alterations. In vivo functional imaging using pan‐neuronal GCaMP lines (Migault et al. 2018; Fornetto et al. 2020) or region‐specific calcium indicators during light–dark and startle paradigms could pinpoint hyper‐ or hypo‐active nuclei. Combining such activity maps with transcriptomic or single‐cell ATAC/RNA profiling in CRISPR mutants (Carey et al. 2025) will help identify downstream gene networks through which *plag1* tunes sensorimotor gating and anxiety‐like behaviour. Taken together, these results suggest that *plag1* function may contribute to the modulation of behavioural responses to sensory stimuli and environmental transitions. Given that *plag1* encodes a zinc finger transcription factor, these behavioural effects are likely to arise through altered regulation of downstream gene networks involved in processes such as synaptic plasticity, stress reactivity, neuronal differentiation and circuit function. Transcriptomic or proteomic profiling of *plag1*‐perturbed larvae will therefore be important for identifying downstream effectors and defining the molecular pathways that underpin altered behavioural adaptation. In parallel, follow‐up behavioural assays, including shoaling preference, social approach/avoidance and conspecific‐cue paradigms, will help determine whether *plag1* also modulates social vigilance or related behaviours relevant to SRS. Together, these complementary molecular, cellular and behavioural approaches will refine our mechanistic understanding of *plag1* in neurodevelopment and may provide a foundation for future therapeutic screening in PLAG1‐associated disorders.

In conclusion, this study establishes a novel role for *plag1* in shaping early behavioural responses in zebrafish. While *plag1*‐perturbation does not grossly impair neurodevelopment, *plag1* disruption alters context‐dependent behavioural outputs in ways that mirror key aspects of SRS. Together with the mild, yet significant alterations in craniofacial development observed in *plag1*‐morphants, our data suggest that the zebrafish is likely to provide a useful model for investigating aspects of PLAG1‐associated SRS phenotypes. These findings position the zebrafish as a valuable genetic tool for dissecting the neural and molecular basis of *PLAG1*‐associated neurodevelopmental phenotypes and lay the foundation for future studies into the molecular and circuit‐level mechanisms underlying its function.

## Author Contributions

All authors (Dr. Jemma G. Gasperoni, Mr. Jarrad N. Fuller, Ms. April L. Lewis, Dr. Travis K. Johnson, Dr. Bert De Groef and Dr. Sebastian Dworkin) contributed to the study conception and design. Material preparation, data collection and data analysis were performed by Dr. Jemma G. Gasperoni, Mr. Jarrad N. Fuller, Ms. April L. Lewis and Dr. Travis K. Johnson and Dr. Bert de Groef provided critical input on data analysis and interpretation. The first draft of the manuscript was written by Dr. Jemma G. Gasperoni and Dr. Sebastian Dworkin. Dr. Travis K. Johnson, Dr. Bert De Groef and Dr. Sebastian Dworkin contributed to manuscript review and revision, and all authors provided comments on, and revisions to, previous versions of the manuscript. All authors read and approved the final manuscript.

## Funding

This work was supported by two separate research grants from the Australian Cranio‐Maxillo‐Facial Foundation (ACMFF; Craniofacial Australia) awarded to A/Prof. Sebastian Dworkin. The funding for these grants was provided to understand the genetic regulation of neurocranial and craniofacial development; the sponsor had no input into the study design, execution, analysis, manuscript conception, planning, writing or decision to publish this work.

## Ethics Statement

All experiments on zebrafish ( *D. rerio* ) were conducted at the La Trobe University under approval granted by the Animal Ethics Committee (AEC) under approved project numbers AEC16‐091 and AEC23‐011.

## Conflicts of Interest

The authors declare no conflicts of interest.

## Supporting information




**Figure S1:** CRISPR/Cas9 targeting and validation of *plag1* in zebrafish crispants. (A) Schematic representation of the *plag1‐201* and *plag1‐202* transcripts and CRISPR guide RNA target sites. Boxes represent protein‐coding and untranslated regions (UTRs), with introns represented by connecting lines. Numbered arrowheads indicate the positions of the four guide RNA target sites used to generate *plag1* crispants. (B–D) Validation of CRISPR/Cas9‐mediated editing by agarose gel electrophoresis of PCR products amplified from genomic DNA of *plag1* crispant and control embryos. Arrowheads indicate additional bands and/or band smearing in crispant samples for guides 1 and 2 (B), guide 3 (C) and guide 4 (D), consistent with insertion/deletion mutations (indels) in mosaic F0 embryos.

**Figure S2:** Behavioural validation of plag1 crispants reveals preserved light–dark responses and acoustic startle habituation. Velocity over time during dark, light and dark periods in control and plag1 crispant larvae (A). Change in swimming distance (Δ) in the first 30 s of light compared with the last 30 s of the preceding dark period (dark‐to‐light transition) (B). A trend towards a reduced response was observed in plag1 crispants (−32.02) compared with controls (−45.99), although this did not reach statistical significance (p = 0.07, Welch's t‐test). Change in swimming distance (Δ) in the first 30 s of dark compared with the last 30 s of the preceding light period (light‐to‐dark transition) (C) was comparable between control (33.31) and plag1 crispant (33.50) larvae (p = 0.98, Welch's t‐test). Maximum velocity (Vmax; mm/s) of control (D) and plag1 crispant (E) larvae across 10 acoustic stimuli with 20‐s interstimulus intervals (ISIs), showing habituation to repeated acoustic stimulation with no significant difference between groups. ‘Resting’ velocity (Vmax) in the 5 s prior to each stimulus (F) was also not significantly different between groups, although plag1 crispants showed a trend towards reduced baseline activity. Error bars represent SEM.

## Data Availability

All data generated or analysed during this study are included in this article (and its Supporting Information). Further enquiries can be directed to the corresponding author.
