# Supplementary material for: Plag1 Regulates Sensorimotor Modulation in Zebrafish
Source: Int J Dev Neurosci. 2026 Oct 2;86(6):e70185. doi: 10.1002/jdn.70185 (PMC13633108; doi:10.1002/jdn.70185)
Supplement: Supplementary file 1 [file JDN-86-0-s001.pdf]

FIGURE S1

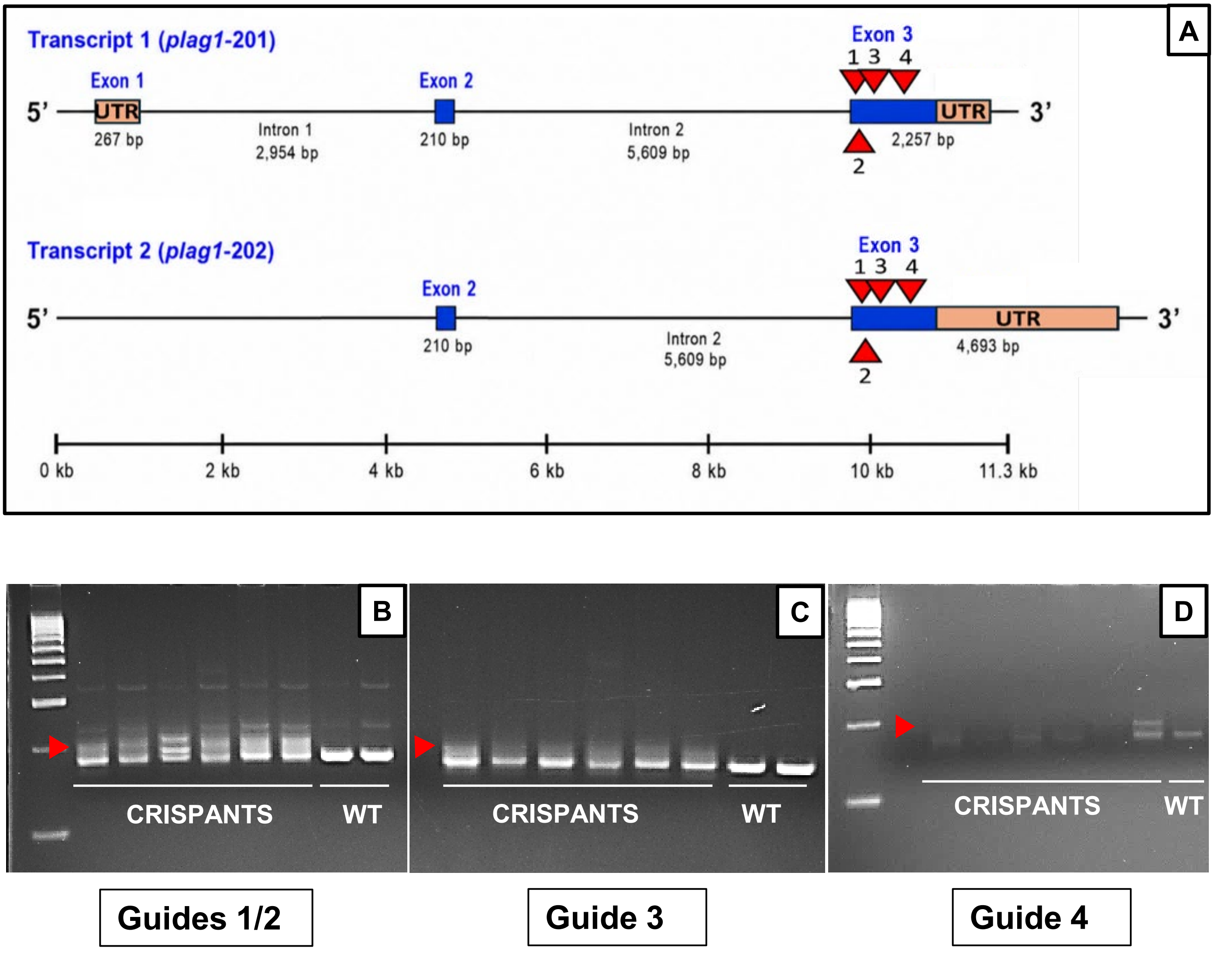
A
Transcript 1 (plag1-201)
Exon 1
Exon 2
Exon 3
1 3 4
5'
UTR
3'
267 bp
Intron 1
2,954 bp
210 bp
Intron 2
5,609 bp
2,257 bp
2
Transcript 2 (plag1-202)
Exon 2
Exon 3
1 3 4
5'
UTR
3'
210 bp
Intron 2
5,609 bp
4,693 bp
2
0 kb
2 kb
4 kb
6 kb
8 kb
10 kb
11.3 kb
B
CRISPANTS
WT
C
CRISPANTS
WT
D
CRISPANTS
WT
Guides 1/2
Guide 3
Guide 4
