# Supplementary material for: Plag1 Regulates Sensorimotor Modulation in Zebrafish
Source: Int J Dev Neurosci. 2026 Oct 2;86(6):e70185. doi: 10.1002/jdn.70185 (PMC13633108; doi:10.1002/jdn.70185)
Supplement: Supplementary file 2 [file JDN-86-0-s002.pdf]

FIGURE S2

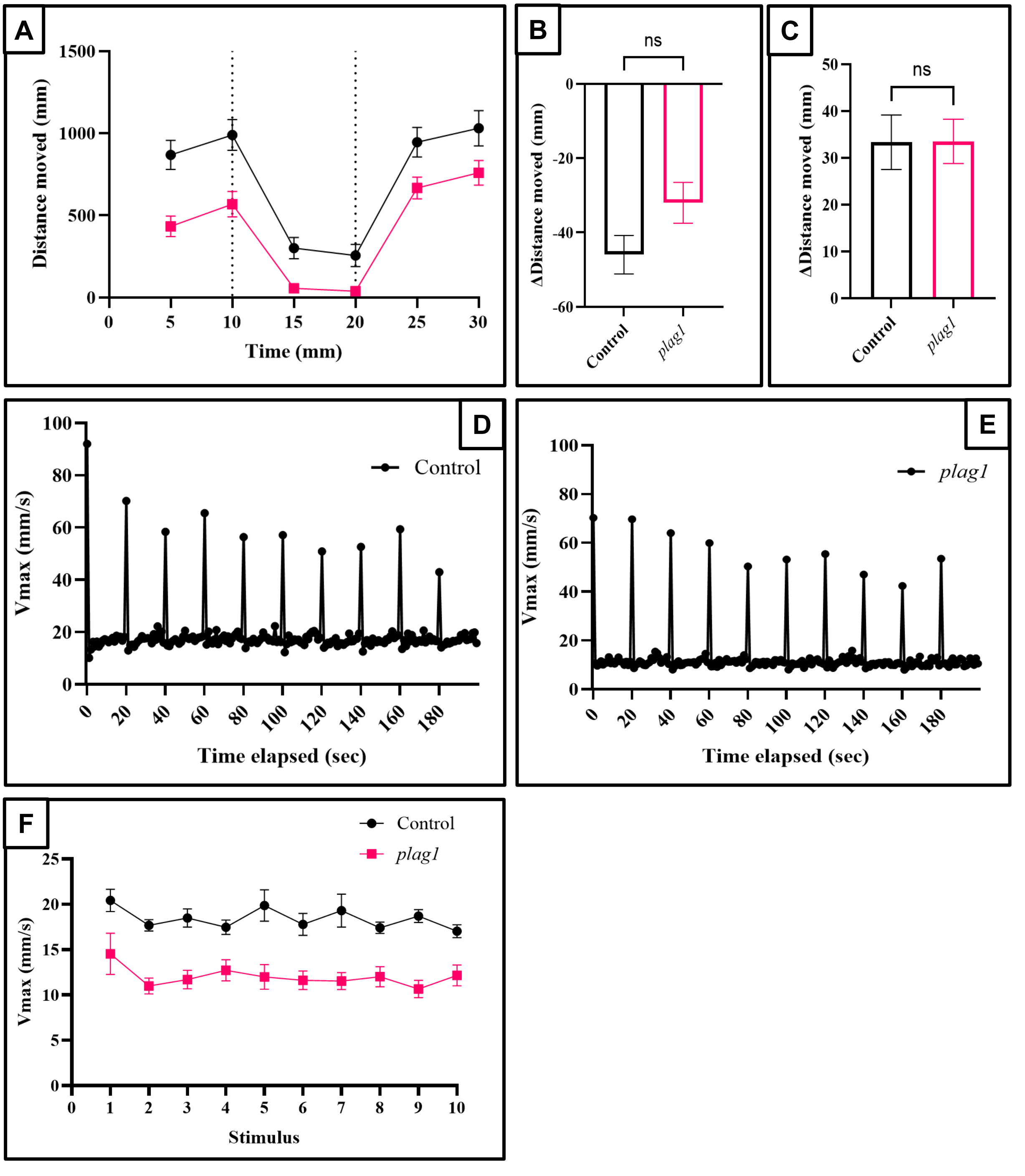
A
Distance moved (mm)
1500
1000
500
0
0
5
10
15
20
25
30
Time (mm)
B
ns
ΔDistance moved (mm)
0
-20
-40
-60
Control
plag1
C
ns
ΔDistance moved (mm)
50
40
30
20
10
0
Control
plag1
D
Control
Vmax (mm/s)
100
80
60
40
20
0
0
20
40
60
80
100
120
140
160
180
Time elapsed (sec)
E
plag1
Vmax (mm/s)
100
80
60
40
20
0
0
20
40
60
80
100
120
140
160
180
Time elapsed (sec)
F
Control
plag1
Vmax (mm/s)
25
20
15
10
5
0
0
1
2
3
4
5
6
7
8
9
10
Stimulus
